# Reclassification and Recombination Analysis of Porcine Epidemic Diarrhea Virus Strains in South Korea Based on Spike Gene Analysis

**DOI:** 10.3390/vetsci13030240

**Published:** 2026-03-01

**Authors:** Eun-Song Lee, Jung-Eun Park

**Affiliations:** College of Veterinary Medicine, Chungnam National University, Daejeon 34134, Republic of Korea; lovelsh0@o.cnu.ac.kr

**Keywords:** porcine epidemic diarrhea virus (PEDV), spike gene, phylogenetic analysis, recombination analysis, South Korea

## Abstract

Porcine epidemic diarrhea (PED) is a severe and highly contagious enteric disease of pigs caused by porcine epidemic diarrhea virus (PEDV). The spike (S) protein of PEDV is the main driving force for viral entry into host cells, influencing the pathogenicity and virulence of the virus subgroup. Currently, the S gene of PEDV exhibits a wide array of variations, with numerous mutations reported. Consequently, recent studies on the phylogenetic classification of PEDV have categorized these variants into clusters or lineages. In this study, 161 PEDV subgroups, including 58 Korean isolates and 103 global PEDV reference subgroups, were classified into two groups, G1 and G2, with nine subgroups (G1a, G1b, G1c, G1d, G2a, G2b, G2c, G2d, and G2e) based on the complete S gene. Phylogenetic analysis revealed that the subgroups isolated in Korea between 2021 and 2022, which were previously reported as G2b subgroups, were G2e subgroups, derived as potential recombinants from the G2a and G1d subgroups. These results indicate that PEDV subgroups isolated from Korea may require reclassification based on recently reported subgroups.

## Simple Summary

Porcine epidemic diarrhea (PED) is a severe, contagious viral disease, causing significant damage to the global swine industry. The PED virus (PEDV) relies on its spike (S) protein for host entry and virulence. However, the S gene undergoes frequent mutations, necessitating updated phylogenetic classifications to track viral evolution accurately. In this study, we analyzed the S genes of 162 sequences, comprising 161 PEDV strains (58 Korean isolates and 103 global references) and one TGEV strain used as an outgroup. The strains were categorized into two major groups (G1 and G2) and nine distinct subgroups (G1a–G1d and G2a–G2e). A critical finding revealed that Korean isolates from 2021 to 2022, previously identified as G2b, actually belong to the G2e subgroup. Evidence suggests these G2e strains are potential recombinants derived from the G2a and G1d subgroups. These results indicate a need to reclassify Korean PEDV isolates to reflect recent genetic shifts and ensure accurate epidemiological monitoring.

## 1. Introduction

Porcine epidemic diarrhea (PED) is a severe and highly contagious enteric disease of pigs caused by porcine epidemic diarrhea virus (PEDV), resulting in watery diarrhea and extreme dehydration. Notably, PEDV can cause up to 100% mortality in newborn piglets without lactogenic immunity [1,2,3]. PEDV is a single-stranded RNA virus of the Coronaviridae family with a positive-sense, enveloped structure [4]. The genome of PEDV is approximately 28 kb long and contains two replicase polyproteins, namely, open reading frame (ORF) 1a and ORF1b; four structural proteins, namely, the spike (S), envelope (E), membrane (M), and nucleocapsid (N) proteins; and one accessory protein, encoded by ORF3 [4].

PEDV can be classified based on each of its encoded genes (ORF1a, ORF1b, ORF3, or N), with the S gene typically used as the basis for phylogenetic analysis and classification [4]. Based on the genetic variability of the S protein, PEDV can be classified into two distinct groups: the classical G1 group and the variant G2 group [4,5]. Generally, the G1 group consists of subgroups, namely, the first identified classical G1a group (CV777, SM98, and virulent DR13) and the G1b group (JS2008, SM-D, and JS-2/2015), while the G2 group comprises the variant subgroups G2a (GD-1, AJ1102, and PEDV JS-A) and G2b (AH2012, GD-B, and GDS25) [6]. According to the current classification, the classical G1a and G1b subgroups are included in the INDEL nomenclature, whereas the G2 group is typically considered non-INDEL [5]. The S-INDEL group includes deletions and insertions in the S gene that emerged in the United States in 2013, currently classified as the G1c (OH851, USA/Ioa106/2013, and MYZ-1/JPN/2013) subgroup [5,7]. Furthermore, G2c (PEDV-LYG, GDS21, and NH-TA2020) and G2d (CH/SCMY/2018, GS2022, and HNJZ-27) subgroups were reported from 2015 to 2023 [6,8,9].

The S protein of coronaviruses plays a crucial role in the production of antibodies, interactions with receptors, and fusion between the viral and cellular membranes [10]. The ectodomain of the S protein consists of the S1 and S2 subunits, with S1 further composed of N-terminal and C-terminal domains (S1-NTD and S1-CTD) [11]. The S1-CTD of PEDV has been suggested to interact with aminopeptidase N (APN) during viral entry [12]. However, recent studies have indicated that the functional role of APN as a PEDV receptor requires further validation and that alternative entry pathways may exist. Therefore, APN should be considered a putative rather than a definitive receptor for PEDV [12]. The S2 region plays a key role in initiating the fusion between the viral envelope and the host cell membrane [11]. Notably, recent phylogenetic approaches have shifted from subgroup-based classifications to more refined analyses based on clusters or lineages, allowing improved resolution for understanding viral evolution and epidemiology. This lineage-based classification provides deeper insights into the genetic relationship between each variant and the transmission patterns of circulating PEDV subgroups, facilitating more targeted vaccine development and disease control strategies [13].

PEDV was initially reported in the late 1970s in the United Kingdom and Belgium, with subsequent outbreaks reported in numerous European countries. In Asia, PEDV was initially discovered in Japan in 1983; since then, China has become a hotspot for PEDV evolution, and the virus has been widely detected in pig farms in Korea and Thailand [14,15,16]. Although PED is prevalent in the majority of these countries, a previous study suggested that its influence on the swine industry did not become substantial until 2010 [17]. Moreover, research has indicated that the virulence of PED was then relatively low in Korea, resulting in minimal negative consequences for pig farming operations [10,18].

The variant PEDV outbreak began in 2010, with the first reported case in the United States occurring in April 2013 and the virus subsequently spreading rapidly throughout the nation [19]. Outbreaks in the United States and various Asian countries were promptly reported, with mortality rates reaching as high as 100% in suckling piglets. Since the end of 2013, a new highly pathogenic mutant of PEDV, classified as G2b, has been shown to cause severe diarrhea and be associated with high mortality rates among Korean piglets [20,21]. Between 2013 and 2017, the S-INDEL PEDV subgroup emerged in Mexico [22]. From 2014 to 2016, the G2a and S-INDEL subgroups were prevalent in Colombia [23]. Among European strains, sporadic cases were reported in Germany, Italy, and Portugal, including a confirmed outbreak in Portugal in 2015 [24]. From 2018 to 2019, PEDV antibodies (in the G2b subgroup) were detected in Vietnam, accompanied by a mutation in the COE neutralizing epitope [25].

PEDV remains a prevalent global issue, with mutations commonly observed. In South Korea, genetic variations, including deletions and insertions in the S gene, have been consistently identified in subgroups up to 2022 [26]. Regarding the situation in Europe, while outbreaks have been sporadic compared to Asia, a recent overview has clarified the current epidemiological landscape and phylogenetic characteristics of PEDV circulating in Europe [2]. Furthermore, comprehensive reviews underscore the ongoing global threat posed by PEDV, discussing its molecular evolution and geographic spread [27].

Currently, the G2b subgroup represents the predominant epidemic genotype of PEDV circulating in South Korea, and conventional vaccine platforms, including inactivated or attenuated G2b-derived formulations, remain the principal means of disease control. Although multiple variants have emerged globally, phylogenetic analyses conducted in South Korea have predominantly classified circulating subgroups into four major subgroups—G1a, G1b, G2a, and G2b—without further exploration into emerging genotypic diversity [26,28,29]. In contrast, neighboring China has expanded its surveillance framework to monitor recombinant PEDV variants, including the G1c, G1d, G2c, and G2d subgroups, with some South Korean isolates even being reclassified under the G2c cluster [6,30].

The emergence of such recombinant subgroups poses a potential risk for South Korea, mirroring the patterns observed in previous cross-border viral introductions. This raises critical questions regarding the continued reliance on G2b-based vaccines, whose efficacy against novel variants remains uncertain. Accordingly, comprehensive molecular investigations are urgently needed to reassess the evolutionary trajectory of PEDV in South Korea and to inform future vaccine design. In this study, we employed bioinformatics approaches to reevaluate the genotypic classification of PEDV subgroups previously designated G2b, aiming to determine whether they represent a divergent or emerging lineage not yet accounted for in the current classification paradigm.

## 2. Materials and Methods

### 2.1. PEDV S Sequences

The complete PEDV S gene was selected for genetic analysis. We obtained 162 sequences from the National Center for Biotechnology Information (NCBI) database. This dataset consisted of 58 Korean field isolates, 103 representative global PEDV reference strains retrieved from GenBank, and one transmissible gastroenteritis virus (TGEV) utilized as an outgroup to root the phylogenetic tree. To ensure a comprehensive comparative analysis, representative global reference strains were selected based on established phylogenetic classification systems described in previous studies [4,6,31,32], encompassing all major G1 and G2 subgroups. The global reference strains were selected for each subgroup: G1a CV777 (GenBank accession no. AF353511), G1b SD-M (GenBank accession no. JX560776), G1c OH851 (GenBank accession no. KJ399978), G1d CH/HNBR/01/2021 (GenBank accession no. MZ161067), G2a AH2012 (GenBank accession no. KC210145), and G2b GD-1 (GenBank accession no. JX647847). Additionally, the G2c strain PEDV-LYG (GenBank accession no. KM609212) isolated in 2015 and the G2d strain CH-SCMY-1-2014 (GenBank accession no. KU975410) isolated in 2017 were included. The Korean isolates analyzed in this study were collected from 2021 to 2022. Detailed information on all selected sequences is provided in Supplementary Table S1.

### 2.2. Multiple Sequence Alignment and Phylogenetic Analysis

For the phylogenetic analysis, we performed multiple sequence alignment of the 162 S sequences and applied the TGEV S sequence (GenBank accession no. AJ271965) as an outgroup. Multiple sequence alignment and phylogenetic analysis were performed via MEGA-X v.12 [33]. The phylogenetic tree was constructed using the neighbor-joining method with the Kimura 2-parameter substitution model, validated by 1000 bootstrap replicates. This method was chosen to ensure consistency with previous epidemiological studies and to facilitate direct comparison of genotyping results. The resulting tree was visualized using Interactive Tree of Life (iTOL) v.7 (https://itol.embl.de/ accessed on 28 January 2026) [34].

### 2.3. Similarity and Recombination Analysis

The Simplot v.3.5.1 program was used to investigate the similarity among the aligned complete PEDV S sequences [35]. All the S sequence data in this study were screened via the Recombination Detection Program version 5 (RDP5); the set for recombination was analyzed using RDP, GENECONV, MaxChi, Chimaera, and 3Seq, followed by secondary scanning and recombination using SiScan and BootScan [36]. Sequences with significant signals for recombination determined by more than two methods were analyzed in greater detail. RDP5 was used with the following parameters (SiScan): window size = 200 bp, step size = 50 bp, and a *p*-value threshold = 0.05. The nucleotide sequence similarity of all the S sequences in this study was detected by SimPlot v.3.5., with a sliding window size of 200 bp, a step size of 50 nucleotides, and 1000 bootstrap replicates, using gap-stripped alignments and the F84 (ML) distance model.

## 3. Results

### 3.1. Phylogenetic Analysis

**Figure 1:**
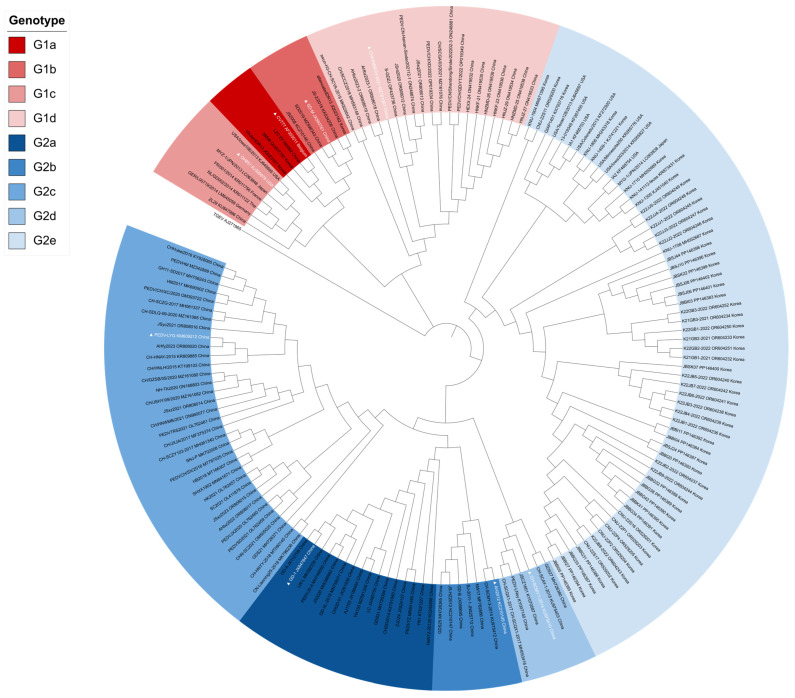
Phylogenetic analysis of PEDV based on nucleotide sequences of the S gene. The phylogenetic tree was constructed with MEGA-X v.11 software using the neighbor-joining method. The reliability of the tree topology was assessed by bootstrap analysis with 1000 replicates. Only bootstrap values greater than 70% are shown at the nodes. The GenBank accession strain names, numbers, and countries are shown in the trees. Different subgroups are indicated by specific colors: G1a (dark red, #C00000), G1b (light red, #E06666), G1c (pink, #EA9999), G1d (pale pink, #F4CCCC), G2a (dark blue, #0B5394), G2b (medium blue, #3D85C6), G2c (sky blue, #6D9EEB), G2d (pale blue, #9FC5E8), and G2e (very pale blue, #CFE2F3). Representatives of each subgroup are highlighted with white font and marked with “▲”.

To investigate the evolutionary relationship between the South Korean PEDV isolates and other PEDV subgroups globally, phylogenetic analyses were performed based on the complete sequences of the S gene of PEDV isolated in South Korea from 2021 to 2022 and a representative reference PEDV subgroup. These PEDVs isolated in South Korea were classified into the G2b subgroup in previous reports [26,28,29]. The phylogenetic tree revealed that the PEDV subgroups clustered into two major groups: G1 and G2 (Figure 1). The G1 group was divided into four subgroups, namely, G1a, G1b, G1c, and G1d, in this study. The G1a subgroup (*n* = 4) included the attenuated vaccine subgroup CV777 and the virulent parental DR13 strain, which formed an independent branch within this subgroup. The G1b subgroup (*n* = 5) contained a cell-culture attenuated DR13 strain and several Chinese field isolates collected since 2012. The G1c subgroup (*n* = 6) included mainly subgroups from 2012 to 2015 and the American strain OH851. The G1d subgroup (*n* = 20) formed an independent branch from the other G1 subgroups and included several subgroups classified as G2d in previous studies. The G2 group was divided into 5 subgroups: G2a, G2b, G2c, G2d, and G2e. The G2a subgroup (*n* = 16) included subgroups isolated in China from 2011 to 2018, and GD-1 was the representative strain of the G2a subgroup. The G2b subgroup (*n* = 22) contained AH2012 and strains isolated in China from 2012 to 2021. The G2c (*n* = 34) and G2d (*n* = 6) subgroups formed an independent branch with the 2017–2023 and 2014–2017 isolates from China, respectively. Subgroups isolated from 2021 to 2022 in South Korea belonged to the G2e subgroup of PEDV. These results indicate that PEDV subgroups isolated in South Korea formed identical clusters with new emerging subgroups from other countries.

### 3.2. Similarity Analysis

**Figure 2:**
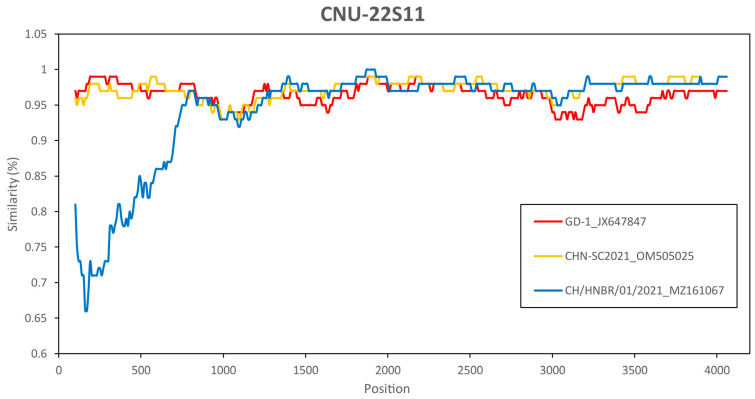
Similarity analysis of the representative strain of PEDV with the CNU-22S11 strain using SimPlot v.3.5.1, window size 200 bp, step size 10, Kimura (2-parameter), and T/t ratio 2.0 condition. The *x*-axis represents the nucleotide positions along the complete spike (S) gene, while the *y*-axis indicates the percentage of nucleotide similarity between the query sequence (CNU-22S11) and reference strains. Colored lines represent comparisons with different reference strains: GD-1 (red), CHN-SC2021 (yellow), and CH/HNBR/01/2021 (blue).

Next, we performed genome-scale similarity comparisons of CNU-22S11 with other PEDV subgroups using SimPlot v3.5.1. This analysis confirmed the significant signal for phylogenetic recombination of at least three subgroups of the CNU-22S11 sample and other subgroups (Figure 2 and Supplementary Figure S1). All the sample data showed high similarity at the front position with the GD-1 and CHN-SC2021 subgroups, but the opposite trend was observed at the middle position with the CH/HNBR/01/2021 subgroup (Supplementary Figure S1).

### 3.3. Recombination Analysis

**Figure 3:**
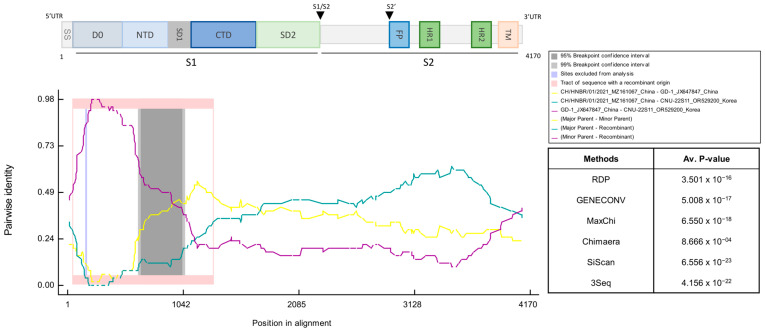
Recombination analysis of the CNU-22S11 S gene. Recombination Detection Program version 5 (RDP5) analysis of the CNU-22S11 strain based on the complete S gene. The *x*-axis indicates nucleotide positions along the alignments, and the *y*-axis represents pairwise identity between the recombinant (CNU-22S11) and reference strains. Colored lines denote sequence identity comparisons between the putative recombinant and parental strains: major parent (CH/HNBR/01/2021, yellow), minor parent (GD-1, purple), and recombinant (CNU-22S11, cyan). Shaded regions indicate 95% (light gray) and 99% (dark gray) breakpoint confidence intervals, while the highlighted tract denotes the genomic region of recombinant origin.

To investigate potential recombination events in the South Korean PEDV isolates, we performed further analysis using the Recombination Detection Program (RDP5). Based on the high sequence similarity observed in the SimPlot analysis, the strains GD-1, CHN-SC-2021, and CH/HNBR/01/2021 were selected as potential parental sequences. The recombination analysis results revealed that the CNU-22S11 subgroup was a recombinant between CH/HNGR.01/2021 (major parent) of the G1d subgroup and GD-1 (minor parent) of the G2a subgroup (Figure 3). This result was supported by more than 5 recombination detection algorithms (Figure 2 and Supplementary Figure S2).

## 4. Discussion

In this study, PEDV subgroups isolated in South Korea were examined in comparison with globally emerging subgroups. The phylogenetic tree constructed with previous reference subgroups, including G1a, G1b, G2a, and G2b, revealed that all the PEDV isolates from South Korea were classified as the G2b subgroup. However, in this paper, the South Korean PEDV isolates were classified into the independent G2e subgroup of PEDV when the G1c, G1d, G2c, and G2d subgroups were included (Figure 1). In this study, we tentatively designate this distinct cluster as the ‘G2e’ subgroup. This nomenclature is proposed to classify the emerging Korean recombinant isolates that are phylogenetically distinct from the established G2a–G2d subgroups, thereby extending the current global classification framework. The sample subgroups isolated in South Korea, which were classified as G2 recombinant subgroups, formed 6 clusters in the refined phylogenetic tree. To investigate whether the isolates classified as the G2e subgroup of PEDV exhibited potential recombination, we conducted similarity analysis with CNU-22S11. The results of the similarity analysis indicated a high degree of similarity at the front position of the sequence in comparison with the GD-1 and CHN-SC2021 strains, but the opposite pattern was observed at the middle position in comparison with the CH/HNBR/01/2021 strain. This indicates the likelihood of a potential recombination event among the sampled subgroups. Next, we used a recombinant detection program to confirm the possibility of recombination. The data show potential recombination, with the GD-1 strain belonging to the G2a subgroup as the minor parent and the CH/HNBR/01/2021 strain belonging to the G1d subgroup as the major parent. The analyzed sample subgroups displayed consistent trends, providing evidence to support the hypothesis that the PEDV isolate from Korea, previously identified as G2b, could be indicative of recombinant variants.

As PEDV variants continue to occur, PEDV classification is becoming increasingly complex and diverse. The occurrence of recombinant variants has been frequently reported in China [6]. Considering the historical precedents of transboundary transmission of swine coronaviruses, there exists a potential risk that these highly pathogenic variants may spread to neighboring countries, potentially precipitating the emergence of novel outbreaks in the region [31]. Therefore, continuous molecular surveillance and improved classification systems are essential for predicting and monitoring the emergence of such high-risk variants. Hence, comprehensive investigations and classification frameworks for subgroups are crucial for accurately predicting and monitoring the transmission pathways of highly pathogenic viruses.

The S protein can trigger the host to create neutralizing antibodies. Six neutralizing epitope regions have been identified in the PEDV S gene, which include the N-terminal domain S10 region (aa 19–220) [37], S1A (aa 435–485) [38], the collagenase equivalent domain (COE) (aa 435–485) [39], SS2 (aa 748–755), SS6 (aa 764–771) [40], and the C-terminal epitope 2C10 (aa 1368–1374) [41]. Crucially, our RDP5 analysis identified a major recombination event spanning amino acid positions 500–1042 of the S protein. This region encompasses key neutralizing epitopes, specifically the COE domain and the SS6 epitope. This suggests that the emergence of the G2e subgroup was driven by the exchange of these critical antigenic determinants via recombination, rather than solely by the accumulation of point mutations. These findings suggest that subgroups isolated in South Korea, including CNU-22S11, have modified S proteins, which are not identical to those of the G2a, G2c, and G2b subgroups classified previously. This may be related to the ongoing occurrence of PEDV in South Korea, despite the application of G2b-based vaccines, but further research is needed.

In addition to phylogenetic and recombination analyses, further investigation of antigenic features is warranted. While detailed residue-by-residue epitope mapping remains to be fully characterized, the observed genetic divergence in the S1 domain, driven by the aforementioned recombination event, suggests that these variants may possess altered antigenic properties compared with classical G2b subgroups. Moreover, the predicted N-linked glycosylation sites were altered in subgroups CNU-22S11 and CNU-22S07, which may impact immune recognition. These findings collectively indicate that antigenic drift in emerging Korean subgroups could influence the antigenicity of current G2b-based vaccines.

The data in this study are valuable for the precise classification of PEDV subgroups currently circulating in South Korea. These findings provide foundational data that could inform future vaccine strain selection strategies for PEDV.

A limitation of this study is that only 58 Korean isolates from the 2021–2022 period were analyzed. Although these sequences represent recent outbreaks, they may not fully reflect the evolutionary dynamics of PEDV in Korea. Incorporating isolates from earlier periods (before 2020) and more recent samples (2023–2025) would provide a more comprehensive overview of viral evolution. Future studies should include a broader temporal dataset to enhance the generalizability of our conclusions.

Although our analyses suggest that the G2e subgroup of the PEDV subgroups may evade the immune response induced by G2b-derived vaccines, this remains a hypothesis. No neutralization assays or amino challenge experiments were conducted in this study. Nevertheless, previous studies have reported immune escape associated with mutations in the COE and SS6 epitopes [39,40]. Thus, the possibility of immune evasion should be considered, although experimental validation is needed.

## 5. Conclusions

In conclusion, based on the S gene analysis, this study suggests that PEDV isolates circulating in South Korea between 2021 and 2022 may belong to a distinct G2e subgroup, likely arising from recombination events between G2a and G1d lineages, rather than the previously identified G2b subgroup. The observed genetic mutations and predicted structural changes within the neutralizing epitopes of the S protein suggest that these variants could possess altered antigenic properties compared to currently used G2b-based vaccines. Consequently, these findings support the refinement of the current phylogenetic classification system to better monitor viral evolution and inform future vaccine strategies. Although these results are based on genomic and structural modeling, further experimental validation and long-term surveillance are warranted to fully understand the evolutionary dynamics and pathogenic potential of these emerging Korean PEDV variants.

## Data Availability

The original contributions presented in this study are included in the article/Supplementary Material. Further inquiries can be directed to the corresponding author.

## Supplementary Materials

The following supporting information can be downloaded at: https://www.mdpi.com/article/10.3390/vetsci13030240/s1, Table S1: The reference strain of S gene information of PEDV. Supplementary Figure S1: Similarity analysis of a representative strain of PEDV with Korean PEDV samples. Supplementary Figure S2: Recombination analysis of the S gene of Korean PEDV samples.

## Abbreviations

The following abbreviations are used in this manuscript:

PED	Porcine Epidemic Diarrhea
PEDV	Porcine Epidemic Diarrhea Virus
RNA	Ribonucleic Acid
ORF	Open Reading Frame
S	Spike
E	Envelope
M	Membrane
N	Nucleocapsid
NTD	N-Terminal Domain
CTD	C-Terminal Domain
APN	Aminopeptidase N
INDEL	Insertions and Deletions
RDP5	Recombination Detection Program Version 5
