# Supplementary material for: Reclassification and Recombination Analysis of Porcine Epidemic Diarrhea Virus Strains in South Korea Based on Spike Gene Analysis
Source: Vet Sci. 2026 Mar 1;13(3):240. doi: 10.3390/vetsci13030240 (PMC13030528; doi:10.3390/vetsci13030240)
Supplement: Supplementary file 1 [file vetsci-13-00240-s001.zip › Supplementary figures.pptx]

## Slide 1
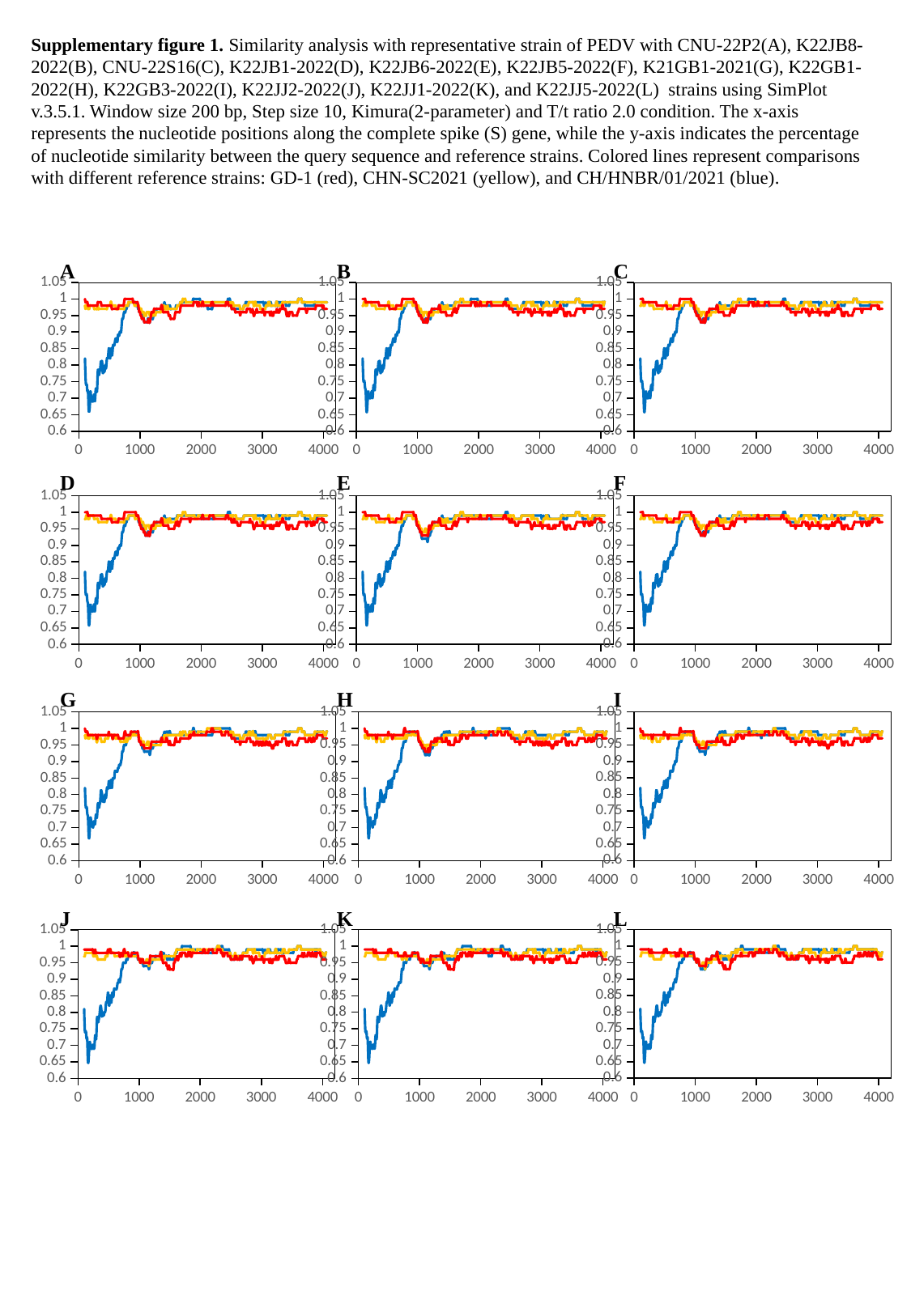

Supplementary figure 1. Similarity analysis with representative strain of PEDV with CNU-22P2(A), K22JB8-2022(B), CNU-22S16(C), K22JB1-2022(D), K22JB6-2022(E), K22JB5-2022(F), K21GB1-2021(G), K22GB1-2022(H), K22GB3-2022(I), K22JJ2-2022(J), K22JJ1-2022(K), and K22JJ5-2022(L) strains using SimPlot v.3.5.1. Window size 200 bp, Step size 10, Kimura(2-parameter) and T/t ratio 2.0 condition. The x-axis represents the nucleotide positions along the complete spike (S) gene, while the y-axis indicates the percentage of nucleotide similarity between the query sequence and reference strains. Colored lines represent comparisons with different reference strains: GD-1 (red), CHN-SC2021 (yellow), and CH/HNBR/01/2021 (blue).
A
B
C
### Chart
| Category | GD-1_JX647847_China | CHN-SC2021_OM505025_China | CH/HNBR/01/2021_MZ161067_China |
|---|---|---|---|
### Chart
| Category | GD-1_JX647847 | CHN-SC2021_OM505025 | CH/HNBR/01/2021_MZ161067 |
|---|---|---|---|
### Chart
| Category | GD-1_JX647847_China | CHN-SC2021_OM505025_China | CH/HNBR/01/2021_MZ161067_China |
|---|---|---|---|D
E
F
### Chart
| Category | GD-1_JX647847_China | CHN-SC2021_OM505025_China | CH/HNBR/01/2021_MZ161067_China |
|---|---|---|---|
### Chart
| Category | GD-1_JX647847_China | CHN-SC2021_OM505025_China | CH/HNBR/01/2021_MZ161067_China |
|---|---|---|---|
### Chart
| Category | GD-1_JX647847_China | CHN-SC2021_OM505025_China | CH/HNBR/01/2021_MZ161067_China |
|---|---|---|---|G
H
I
### Chart
| Category | GD-1_JX647847_China | CHN-SC2021_OM505025_China | CH/HNBR/01/2021_MZ161067_China |
|---|---|---|---|
### Chart
| Category | GD-1_JX647847_China | CHN-SC2021_OM505025_China | CH/HNBR/01/2021_MZ161067_China |
|---|---|---|---|
### Chart
| Category | GD-1_JX647847_China | CHN-SC2021_OM505025_China | CH/HNBR/01/2021_MZ161067_China |
|---|---|---|---|J
K
L
### Chart
| Category | GD-1_JX647847_China | CHN-SC2021_OM505025_China | CH/HNBR/01/2021_MZ161067_China |
|---|---|---|---|
### Chart
| Category | GD-1_JX647847_China | CHN-SC2021_OM505025_China | CH/HNBR/01/2021_MZ161067_China |
|---|---|---|---|
### Chart
| Category | GD-1_JX647847_China | CHN-SC2021_OM505025_China | CH/HNBR/01/2021_MZ161067_China |
|---|---|---|---|

## Slide 2
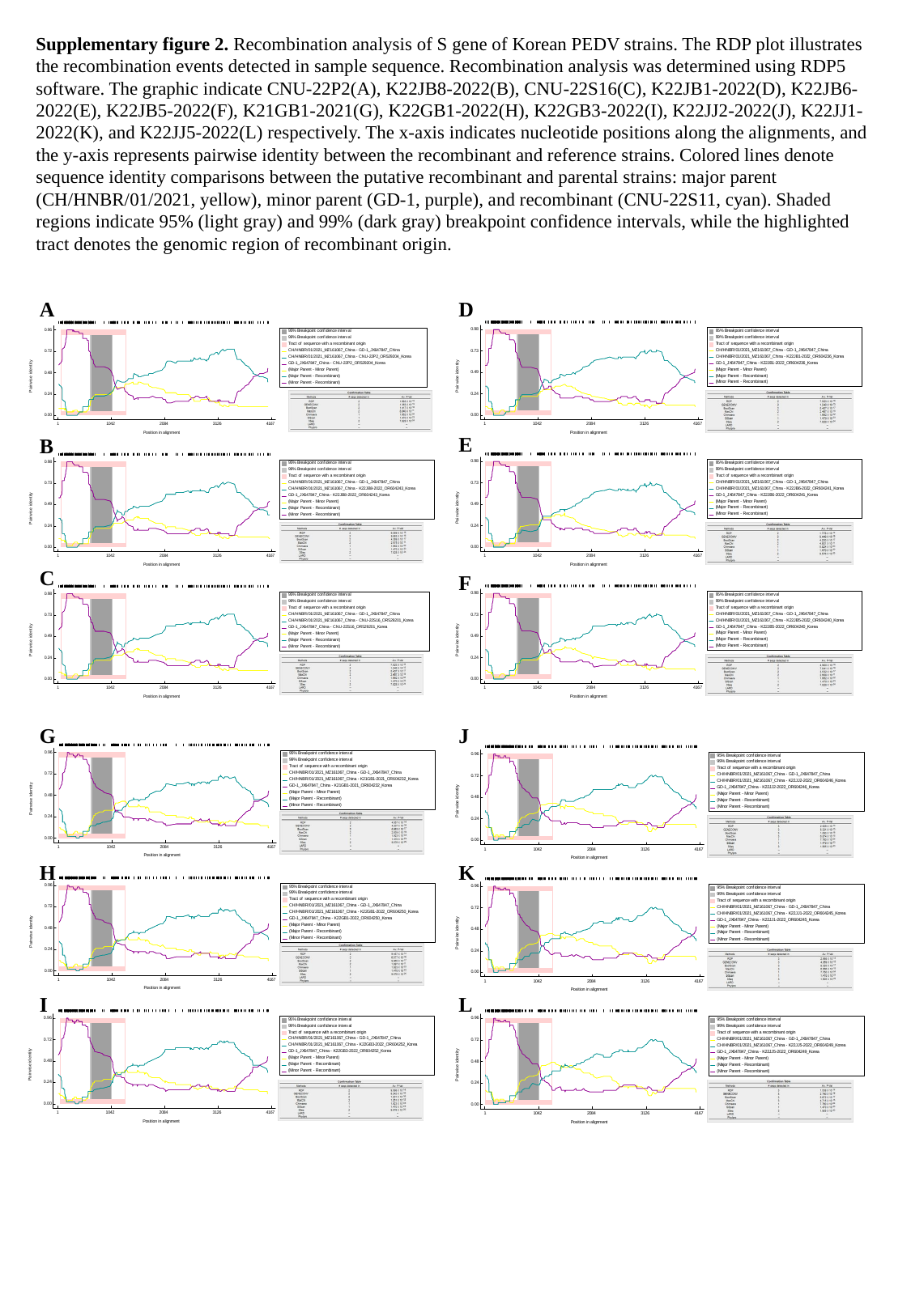

Supplementary figure 2. Recombination analysis of S gene of Korean PEDV strains. The RDP plot illustrates the recombination events detected in sample sequence. Recombination analysis was determined using RDP5 software. The graphic indicate CNU-22P2(A), K22JB8-2022(B), CNU-22S16(C), K22JB1-2022(D), K22JB6-2022(E), K22JB5-2022(F), K21GB1-2021(G), K22GB1-2022(H), K22GB3-2022(I), K22JJ2-2022(J), K22JJ1-2022(K), and K22JJ5-2022(L) respectively. The x-axis indicates nucleotide positions along the alignments, and the y-axis represents pairwise identity between the recombinant and reference strains. Colored lines denote sequence identity comparisons between the putative recombinant and parental strains: major parent (CH/HNBR/01/2021, yellow), minor parent (GD-1, purple), and recombinant (CNU-22S11, cyan). Shaded regions indicate 95% (light gray) and 99% (dark gray) breakpoint confidence intervals, while the highlighted tract denotes the genomic region of recombinant origin.
A
D
E
B
C
F
G
J
H
K
I
L
